# Pandemic one health clones of *Escherichia coli* and *Klebsiella pneumoniae* producing CTX-M-14, CTX-M-27, CTX-M-55 and CTX-M-65 ESβLs among companion animals in northern Ecuador

**DOI:** 10.3389/fcimb.2023.1259764

**Published:** 2025-01-07

**Authors:** Fernando A. Gonzales-Zubiate, José Humberto M. Tambor, Juan Valencia-Bacca, María Fernanda Villota-Burbano, Adriana Cardenas-Arias, Fernanda Esposito, Quézia Moura, Bruna Fuga, Elder Sano, Jesus G. M. Pariona, Mishell Poleth Ortiz Jacome, Nilton Lincopan

**Affiliations:** ^1^ School of Biological Sciences & Engineering, Yachay Tech University, San Miguel de Urcuquí, Ecuador; ^2^ Centro Universitário ENIAC, São Paulo, Brazil; ^3^ INTI International University, Persiaran Perdana BBN, Nilai, Negeri Sembilan, Malaysia; ^4^ Department of Microbiology and Immunology, Wake Forest School of Medicine, Winston Salem, NC, United States; ^5^ Dogs & Cats Hospital Veterinario, Ibarra, Imbabura, Ecuador; ^6^ One Health Brazilian Resistance Project (OneBR), São Paulo, Brazil; ^7^ Department of Microbiology, Institute of Biomedical Sciences, University of São Paulo, São Paulo, Brazil; ^8^ Department of Clinical Analysis, School of Pharmacy, University of São Paulo, São Paulo, Brazil; ^9^ Federal Institute of Espírito Santo, Vila Velha, Brazil

**Keywords:** ESβL, gram-negative bacteria, Enterobacterales, antimicrobial resistance, One Health, veterinary medicine, genomic data

## Abstract

From a One Health perspective, dogs and cats have begun to be recognized as important reservoirs for clinically significant multidrug-resistant bacterial pathogens. In this study, we investigated the occurrence and genomic features of ESβL producing Enterobacterales isolated from dogs, in the province of Imbabura, Ecuador. We identified four isolates expressing ESβLs from healthy and diseased animals. In this regard, two *Escherichia coli* strains producing CTX-M-55-like or CTX-M-65 ESβLs belonged to the international ST10 and ST162, whereas two *Klebsiella pneumoniae* producing CTX-M-14 or CTX-M-27 belonged to ST35 and ST661. Phylogenomic analysis clustered (95-105 SNP differences) CTX-M-55/ST10 *E. coli* from companion animal with food and human *E. coli* strains of ST10 isolated in 2016, in Australia and Cambodia, respectively; whereas CTX-M-27-positive *K. pneumoniae* ST661 was clustered (201-216 SNP differences) with human strains identified in Italy, in 2013 and 2017, respectively. In summary, we report the presence and genomic data of global human-associated clones of CTX-M-producing *E. coli* and *K. pneumoniae* in dogs, in Ecuador. The implementation of a national epidemiological surveillance program is necessary to establish future strategies to control the dissemination of antibiotic-resistant priority pathogens using a One Health approach.

## 1 Introduction

Although Enterobacterales are natural inhabitants of the intestinal tract of mammals, some genus and species can cause infections of the respiratory and urinary systems, skin, ear, and soft tissue of human and non-human hosts (Zogg et al., 2018). In this regard, *Escherichia coli* and *Klebsiella pneumoniae* are leading causes of healthcare-associated infections worldwide (Pesesky et al., 2015), with carbapenem- and broad-spectrum cephalosporin-resistant lineages being categorized as critical priority pathogens by the World Health Organization (Tacconelli et al., 2018). Certainly, and even more worrying is the fact that extended-spectrum β-lactamase (ESβL)-producing strains are no longer restricted to hospital locations but also represents a serious problem involving pets, wildlife, and environmental and food safety (Lopes et al., 2021; Salgado-Caxito et al., 2021).

CTX-M enzymes have become the most prevalent type of ESβLs globally (Cantón & Coque, 2006; Pitout and Laupland, 2008). It is remarkable that the first report on the emergence of a CTX-M enzyme was in 1988, from a laboratory dog used in β-lactams research in Japan (Matsumoto et al., 1988), whereas *E. coli* producing *bla*_CTX-M-1_-type enzyme was first described in a healthy dog in Portugal. Since then, a significant occurrence of CTX-M-type ESβL-producing Enterobacterales has been documented in healthy and diseased dogs and cats from Asian, European and South American countries (Salgado-Caxito et al., 2021).

From a public health perspective, the rapid appearance of resistant bacterial populations among dogs and cats, and the close contact between household pets and people have favored the transmission of antibiotic-resistant bacteria from companion animals to humans (Damborg et al., 2016; Kawamura et al., 2017; Salgado-Caxito et al., 2021; Sellera et al., 2021). Transfer of resistant bacteria between humans and their dogs has been well documented (Albrechtova et al., 2012), as was illustrated by the identification of the same *E. coli* clone from a urinary tract infection in a dog, and from its household members (Johnson et al., 2008), although the direction of transfer is often difficult to prove (Pomba et al., 2017). In addition, the intensive use of antimicrobials in animals can be an important factor in the development of antimicrobial-resistant microorganisms (Caprioli et al., 2000; Umber and Bender, 2009; Marshall and Levy, 2011; Seiffert et al., 2013; Samanta et al., 2015). In this sense, companion animals might act as source of human contamination but may also be contaminated by human bacteria (Okubo et al., 2014; Fernandes et al., 2018; Melo et al., 2018). Furthermore, the role of companion animals as a source of AMR has, so far, been neglected (Ewers et al., 2012).

In South America, multidrug-resistant Enterobacterales are a major concern as the region exhibits some of the higher rates of antimicrobial resistance worldwide (Bonelli et al., 2014). The first report of ESβL in this region in companion animals was published in 2008 from *E. coli* isolates obtained from fecal samples of dogs and cats in Chile (Moreno et al., 2008). In that context, nosocomial infections caused by ESβL producing Enterobacterales have increased in the region more than others, since 2005 (Guzmán-Blanco et al., 2014). Several factors such living in crowded conditions, malnutrition, ineffective healthcare systems, deficient drug supply chain, massive use of antimicrobials in livestock and agriculture linked to lack of financial resources might be related to the greater prevalence of ESβLs in countries with lower economic resources (Villegas et al., 2008). In this study, we report the occurrence and genomic data of ESβL-producing *E. coli* and *K. pneumoniae* strains in dogs from Imbabura, Ecuador.

## 2 Materials and methods

### 2.1 Bacterial isolates and antibiotic susceptibility profile

During a microbiological and genomic surveillance study carried out in 2018, a total of 125 rectal swabs from dogs (64 healthy animals and 61 sick animals) were collected from the province of Imbabura in Ecuador, in order to monitor the presence of clinically significant drug-resistant Gram-negative bacteria in companion animals (**Supplementary Table 1**). Samples were collected between April and June and between October and December 2018; from a veterinary clinic located in Ibarra that attend the following counties in Imbabura: Antonio Ante, Cotacachi, Ibarra, Otavalo, Pimampiro, and San Miguel de Urcuquí (**Figure S1**).

The samples were cultured on blood and MacConkey agar plates supplemented with ceftriaxone (2 µg/mL) being incubated at 37°C overnight (Jacob et al., 2020). Bacteria were identified by conventional biochemical tests, whereas antimicrobial susceptibility testing was performed by the disk diffusion method on Mueller–Hinton agar plates (Clinical and Laboratory Standards Institute, 2023a; Clinical and Laboratory Standards Institute, 2023b). In addition, human and veterinary antibiotics including amoxicillin-clavulanic acid, ceftazidime, cefotaxime, ceftriaxone, ceftiofur, cefepime, cefoxitin, aztreonam, ertapenem, meropenem, imipenem, nalidixic acid, enrofloxacin, ciprofloxacin, trimethoprim/sulfamethoxazole, gentamicin, amikacin, and chloramphenicol, were tested (**Supplementary Table 2**). Additionally, minimum inhibitory concentration (MIC) of cefotaxime was determined by using ETEST® strips (bioMérieux). The results were interpreted according to Clinical and Laboratory Standards Institute (Clinical and Laboratory Standards Institute, 2023a; Clinical and Laboratory Standards Institute, 2023b). ESβL production was screened by the double disk synergy test (DDST) (Jarlier et al., 1988).

### 2.2 Whole genome sequencing analysis

Whole genomic DNA was extracted (PureLinkTM; Invitrogen) and used to prepare a library that was sequenced using the NextSeq550 platform (2 x 75-bp paired-end) (Illumina), and the *de novo* assembly method was the Unicycler v.0.4.8 with Phred20 as minimum score quality of reads. The contigs generated for all genomes were submitted to NCBI using the WGS submission and automatic annotation was performed by PGAP (Prokaryotic Genome Annotation Pipeline v.3.2.); CDSs, RNAs and pseudo genes are shown in **Tables 1**, **2**. The genomes were analyzed by MLST 2.0, ResFinder 4.1, and PlasmidFinder 2.1 tools from the Center for Genomic Epidemiology (CGE). Additionally, antibiotic resistance and virulence genes were predicted using the Comprehensive Antibiotic Resistance Database (CARD) and the Virulence Factor Database (VFDB), respectively, whereas genes related with mercury, arsenic and disinfectant resistance (quaternary ammonium compounds) were screened using an in-house and the BIGSdb database. For phylotyping *E. coli*, the *in silico* Clermont phylotyper tool was used (https://ezclermont.hutton.ac.uk/).

**Table 1:** Genomic characteristics of lineages of ESBL-producing *Escherichia coli* strains recovered from rectal swabs collected in dogs in Ecuador.

Characteristics	ECU3_SQ178	EE12_SQ154
Source	Dog rectal swab	Dog rectal swab
Year of isolation	2018	2018
Genome size (bp)	4,847,206	4,893,054
G + C content (%)	50,7	50,7
rRNA	2	2
tRNAs	45	39
ncRNAs	7	9
N° total of genes	4,784	4,727
No. of CDS^a^	4,595	4,549
ST	10	162
Clermont phylotype	A	B1
Resistome		
* β*-Lactams	*bla*_CTX-M-55_-like	*bla*_CTX-M-65_
Aminoglycosides	*aph(3’’)-Ib*, *aph(6)-Id*	*aadA1*, *aadA2b*
Phenicols	*florR*	*clmA1*
Sulfonamides	*sul2*	*sul3*
Tetracycline	*tetA*	–
Trimethoprim	–	*dfrA1*
Fosfomycin	*fosA3*	*fosA3*
Quinolones	*gyrA* (D87N), *parC* (S80I), *marA*	*marA*
Heavy metal		
* *Arsenic	*arsB*, *arsC, arsR*	*arsB*, *arsR*
* *Mercury	*-*	*merR*
* *Tellurium	*tehA*, *tehB*	*tehA*, *tehB*
Biocides and disinfectants	*mdtEFKN, emrDK*, *acrAEF*, *tolC*	*mdtEFK*, *emrDK, mvrC*, *acrAEF*, *tolC*, *qacF*
Herbicides (glyphosate)	*phnCDFGHIJKLMNOP*	*phnJ*
Virulome		
Common pilus	*yagZ/ecpA, yagY/ecpB, yagX/ecpC, yagW/ecpD*	*yagZ/ecpA*, *yagY/ecpB*, *yagX/ecpC*, *yagW/ecpD*
Fimbrial protein	*-*	*fimBCDEGI*
Enterobactin siderophore	*entB*	*-*
Salmochelin siderophore	*iroCDEN*	*iroCDEN*
Type II secretion system (T2SS)	*gspM*	*gspK*
Plasmids	IncFIA, IncFIB, IncFII	IncFIB
GenBank accession number	JACWHI000000000.1	JACWHK000000000.1

**Table 2:** Genomic characteristics of lineages of ESBL-producing *Klebsiella pneumoniae* strains recovered from rectal swabs collected in dogs in Ecuador.

Characteristics	ECUD12_SQ166	EE25K_SQ190
Source	Dog rectal swab	Dog rectal swab
Year of isolation	2018	2018
Genome size (bp)	5,342,763	5,560,571
G + C content (%)	57,4	57,2
rRNA	2	2
tRNAs	39	46
ncRNAs	7	8
N° total of genes	5,283	5,412
No. of CDS^a^	5,142	5,270
ST	661	35
K-locus/O-locus	KL28/O2v1	-/O1v1
*wzi/*ICE*Kp*/*ybt*	84/-/-	37/ICE*Kp*3/*ybt 9*
Resistome		
* β*-Lactams	*bla*_CTX-M-27_, *bla*_SHV-27_	*bla*_CTX-M-14_, *bla*_LAP-2_, *bla*_SHV-33_
Aminoglycosides	*aac(3)IV*, *aac(6′)-Ib-cr*, *aadA1*, *aadA16*, *aadA2b*, *aph(4)-la*	–
Phenicols	*clmA1*	–
Sulfonamides	*sul1*, *sul3*	*sul1*
Tetracycline	*tetD*	–
Trimethoprim	*dfrA27*	*dfrA1*
Fosfomycin	*fosA6*	*fosA6*
Quinolones	*qnrB52*, *aac(6’)-Ib-cr*, *oqxA*, *oqxB*	*qnrS1*, *oqxA*, *oqxB*
Macrolides	*mphA*	–
Rifampicin	*arr-3*	–
Heavy metal		
* *Arsenic	–	*arsB, arsC, arsD, arsR*
* *Silver	*silABCEFRS*	*silABCEFRS*
Biocides and disinfectants	*qacF*	*smvR*
Virulome		
Yersiniabactin siderophore	–	*ybtSXQPAUTE*, *irp1*, *irp2*, *fyuA*
Plasmids	IncFIB	IncFIB
GenBank accession number	JACWHJ000000000.1	JACWHL000000000.1

### 2.3 Phylogenetic analysis

A search for genomic data of isolates for each sequence type identified was performed, in order to recruit genomes for phylogenetic comparison. Assemblies with no metadata for country, year and source of isolation were ignored. For *E. coli* strains, genomes were downloaded from Enterobase (3,572 assemblies of *E. coli* ST10 and 442 assemblies of ST162), while for *K. pneumoniae* strains, a search for each ST were performed on bacWGSTdb (http://bacdb.cn/BacWGSTdb/), and genomes were downloaded from NCBI GenBank (*i.e*., 60 assemblies of *K. pneumoniae* ST35 and 19 assemblies of ST661). With exception of ST661, which had only 19 assemblies downloaded, 30 genomes with highest average nucleotide identity (ANI) of each ST comparing with this work’s assemblies were performed using FastANIv1.32 (https://github.com/ParBLiSS/FastANI/). ANI values between downloaded and query genomes were ≥99.7625% for *E. coli* ST10, ≥99.7807% for *E. coli* ST162, ≥99.7631% for *K. pneumoniae* ST35 and ≥99.575% for *K. pneumoniae* ST661. CSI Phylogeny (https://cge.food.dtu.dk/services/CSIPhylogeny/) was used with default settings to generate approximate maximum-likelihood SNP-based trees. Chromosome sequences of SCU-118 (NZ_CP051716.1) and LD91-1 (NZ_CP042585.1) *E. coli* strains, and RJY9645 (NZ_CP041353.1) and F13 (NZ_CP026162.1) of *K. pneumoniae* strains were used as reference for *E. coli* ST10 and ST162, and *K. pneumoniae* ST35 and ST661, respectively. ABRicatev1.0.1 (https://github.com/tseemann/abricate) was used with ResFinder and PlasmidFinder databases to screen antimicrobial resistance genes and plasmids on each recruited genome. Identity and coverage limits were set to 98% and 100%, respectively. iTOLv6 (https://itol.embl.de/) was used to annotate the tree with data from Enterobase, bacWGSTdb and ABRicate.

## 3 Results and discussion

Forty-tree cephalosporin-resistant Gram-negative bacteria were isolated from 23 healthy dogs and 16 sick dogs (**Supplementary Tables 1, 2**). From the latter, eight dogs presented with gastrointestinal complications, four with metabolic syndrome, two with dermatological disease, one with respiratory problems, and another with cerebrovascular accident. Based on confirmation of ESβL phenotype, four bacterial isolates exhibiting a MDR profile (Magiorakos et al., 2012) were sequenced: i) *E*. *coli* strain ECU3_SQ178 (GenBank accession number: JACWHI000000000.1) isolated from a 6-month-old healthy female dog mixed breed, with no previous treatments reported. This strain presented resistance to ceftazidime, cefotaxime (MIC > 32 µg/mL), ceftriaxone, cefepime, aztreonam, nalidixic-acid, enrofloxacin, ciprofloxacin, and chloramphenicol, being susceptible to amoxicillin-clavulanic acid, cefoxitin, ertapenem, meropenem, imipenem, gentamicin, amikacin, trimethoprim-sulfamethoxazole (**Supplementary Table 2**). In this regard, WGS analysis predicted the presence of genes associated with resistance to β-lactams (*bla*_CTX-M-55_-like), phenicols (*floR*), tetracyclines (*tetA*), sulphonamides (*sul2*), aminoglycosides [*aph(3”)-Ib*, *aph(6)-Id*], fosfomycin (*fosA3*), and quinolones (*gyrA-*D87N and *parC*-S80I point mutations, *marA*). On the other hand, genes conferring tolerance to heavy metals [arsenic (*arsBCR*) and tellurium (*tehAB*)], herbicide [glyphosate (*phnCDFGHIJKLMNOP*)], biocides and disinfectants (*mdtEFKN*, *emrDK*, *acrAEF* and *tolC*) were also predicted (**Table 1**); ii) *E. coli* strain EE12_SQ154 (GenBank accession number: JACWHK000000000.1) isolated from a 4-years-old female Yorkshire terrier dog with a history of physical decline, cerebrovascular accident and shock. It was not reported by the private veterinary clinic the treatment received prior to the sample collection. Antimicrobial susceptibility testing revealed resistance to ceftazidime, cefotaxime (MIC > 32 µg/mL), ceftriaxone, cefepime, aztreonam, nalidixic-acid, enrofloxacin, ciprofloxacin, trimethoprim-sulfamethoxazole, chloramphenicol, and gentamicin, and susceptibility to amoxicillin-clavulanic acid, cefoxitin, ertapenem, meropenem, imipenem and amikacin (**Supplementary Table 2**). The antimicrobial resistome included genes conferring resistance to β-lactams (*bla*_CTX-M-65_), aminoglycosides (*aadA1*, *aadA2b*), fosfomycin (*fosA3*), phenicol (*cmlA1*), sulphonamides (*sul3*), trimethoprim (*dfrA1*), quinolones (*marA*), heavy metals [arsenic (*arsBR*), tellurium (*tehAB*) and mercury (*merR*)], herbicide [glyphosate (*phnJ*)], biocides and disinfectants (*mdtEFK*, *emrDK*, *acrAEF*, *tolC*, *qacF* and *mvrC*) (**Table 1**); iii) *K. pneumoniae* strain ECU12_SQ166 (GenBank accession number: JACWHJ000000000.1), isolated from a 12-year-old male English Shepherd dog admitted to a private veterinary clinic with signs of diarrhea, melena, vomiting, septicemia, and chronic kidney failure leading to death. Based on the anamnesis and initial physical examination, fluid therapy was established, as a stabilization measure (lactated ringer solution), and a not specified β-lactam antibiotic was administered. The strain exhibited resistance to amoxicillin-clavulanic acid, ceftazidime, cefotaxime (MIC > 32 µg/mL), ceftriaxone, cefepime, aztreonam, ceftiofur, trimethoprim-sulfamethoxazole, nalidixic-acid, enrofloxacin, ciprofloxacin, and gentamicin, being susceptible to cefoxitin, ertapenem, meropenem, imipenem, and amikacin (**Supplementary Table 2**). The resistome analysis predicted resistance genes to β-lactams (*bla*_CTX-M-27_, *bla*_SHV-27_), fosfomycin (*fosA6*), trimethoprim (*dfrA27*), rifampicin (*arr-3*), sulfonamides (*sul1*, *sul3*), aminoglycosides [*aac(3)IV, aac*(*6′*)*-Ib-cr*, *aadA1*, *aadA16*, *aadA2b*, *aph(4)-la*], macrolides (*mphA*), quinolones [*aac(6’)-Ib-cr*, *oqxA, oqxB, qnrB52*], phenicols (*cmlA*), tetracyclines (*tetD*), silver (*silABCEFRS*) and ammonium quaternary compounds (*qacF*) (**Table 2**); (iv) *K. pneumoniae* strain EE25K_SQ190 (GenBank accession number: JACWHL000000000.1) isolated from an 8-year-old male German shepherd dog, presenting with discomfort, anorexia and foreign body gingivitis. After clinical examination, the foreign body was removed and a combination of amoxicillin/clavulanic acid plus a non-steroidal anti-inflammatory was prescribed. Antimicrobial susceptibility testing revealed resistance to cefotaxime (MIC > 32 µg/mL), ceftriaxone, cefepime, nalidixic-acid, enrofloxacin, ciprofloxacin, and trimethoprim-sulfamethoxazole. This strain showed to be susceptible to amoxicillin-clavulanic acid, ceftazidime, cefoxitin, aztreonam, ertapenem, meropenem, imipenem, amikacin and gentamicin (**Supplementary Table 2**). Resistome encompass genes resistant to β-lactams (*bla*_CTX-M-14_, *bla*_SHV-33_, *bla*_LAP2_), fosfomycin (*fosA6*), trimethoprim (*dfrA1*), quinolones (*oqxA, oqxB, qnrS1*), and sulphonamides (*sul1*), silver (*silABCEFRS*), arsenic (*arsBCDR*) and chlorhexidine (*smvR*) (**Table 2**).

**Figure 1:**
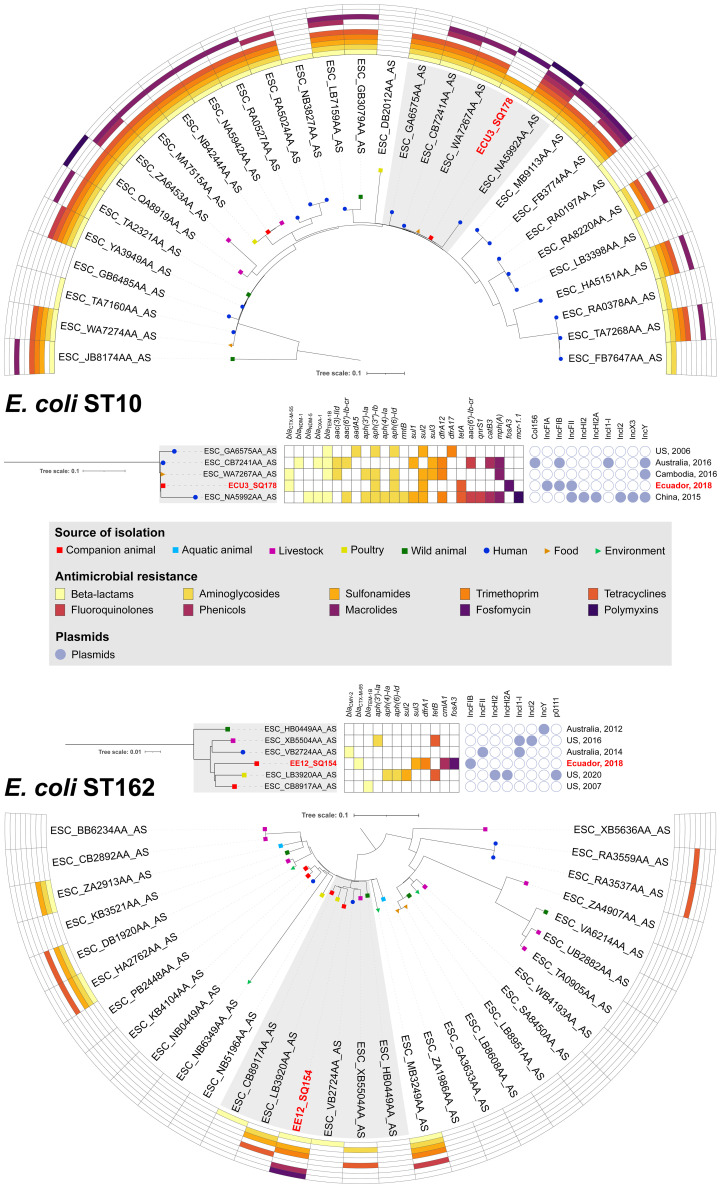
Phylogenetic trees of *E. coli* strains. SNP-based phylogenetic trees of *E. coli* ST10 and ST162, and heatmap showing presence/absence of antibiotic resistance genes for 10 antibiotics, and their source of isolation. Details on resistome, plasmidome and origin are showed for clusters formed with CTX-M-55-producing *E. coli* ECU3_SQ178 and CTX-M-65 *E. coli* EE12_SQ154 strains, isolated from dogs, in Ecuador.

While CTX-M-55- and CTX-M-65-positive *E. coli* strains belonged to ST10 and ST162, *K. pneumoniae* producing CTX-M-27 and CTX-M-14 ESβLs belonged to ST661 and ST35, respectively. *E. coli* ST10 and ST162 have been previously associated with human infections (Coelho et al., 2011; Chen et al., 2014), being further identified in hospital sewage (Zhao et al., 2017), bovines (Umpiérrez et al., 2017), birds (Fuentes-Castillo et al., 2020), and dogs (Yasugi et al., 2021). The *bla*_CTX-M-55_ gene has been widely identified globally in *E. coli* isolates from various animal species (Kiratisin et al., 2007; Zhang et al., 2014; Birgy et al., 2018; Lupo et al., 2018). The remarkable prevalence of this gene, accompanied by a high propensity for horizontal gene transfer has facilitated its rapid and wide spread (Yang et al., 2023). In Ecuador *bla*_CTX-M-55_ has been the most prevalent allele of the *bla*_CTX-M_ family in *E. coli* from poultry settings, followed by *bla*_CTX-M-65_ and *bla*_CTX-M-2_ (Ortega-Paredes et al., 2020a). On the other hand, according to Enterobase (https://enterobase.warwick.ac.uk/), ST10 has been identified in dogs from Germany, United States of America (USA), United Kingdom, South Korea, Canada and New Zealand, whereas in Ecuador ST10 has been identified in humans, wild animals, and environmental samples; confirming the One Health importance of this global lineage in this country. In fact, phylogenomic analysis showed that strain ECU3_SQ178 (CTX-M-55/ST10) clustered (95-105 SNP differences) with food and human *E. coli* strains of ST10 isolated in 2016, in Australia and Cambodia, respectively, whereas CTX-M-65-positive *E. coli* ST162 (strain EE12_SQ154) showed ubiquity, being clustered (207-265 SNP differences) with other four drug-resistant *E. coli* strains of ST162 isolated from livestock (USA, 2016), poultry (USA, 2020), human (Australia, 2014) and companion animal (USA, 2007) (**Figure 1**, **Supplementary Table 3**). Moreover, data retrieved from Enterobase confirm occurrence of this *E. coli* clone in companion animals from Germany, USA, and Canada. Interestingly, this is the first report of *E. coli* ST162 found in companion animal, in South America.

In the case of *K. pneumoniae* ST661 and ST35 clones, they have been previously isolated from nosocomial pneumonia in humans (Zhao et al., 2019), rectal swabs from pigs and fecal human samples (Leangapichart et al., 2021). Moreover, ST661 has been recovered from aquatic environments (Furlan et al., 2020), hospitalized patients (Piazza et al., 2019), being recently reported as responsible for outbreaks in Europe (Martin et al., 2017); whereas ST35 has been identified among ESβL-producing *K. pneumoniae* strains in hospital settings (Marcade et al., 2013; Frenk et al., 2020), being lately recognized as a multidrug-resistant clone with worldwide distribution (Shen et al., 2020).

**Figure 2:**
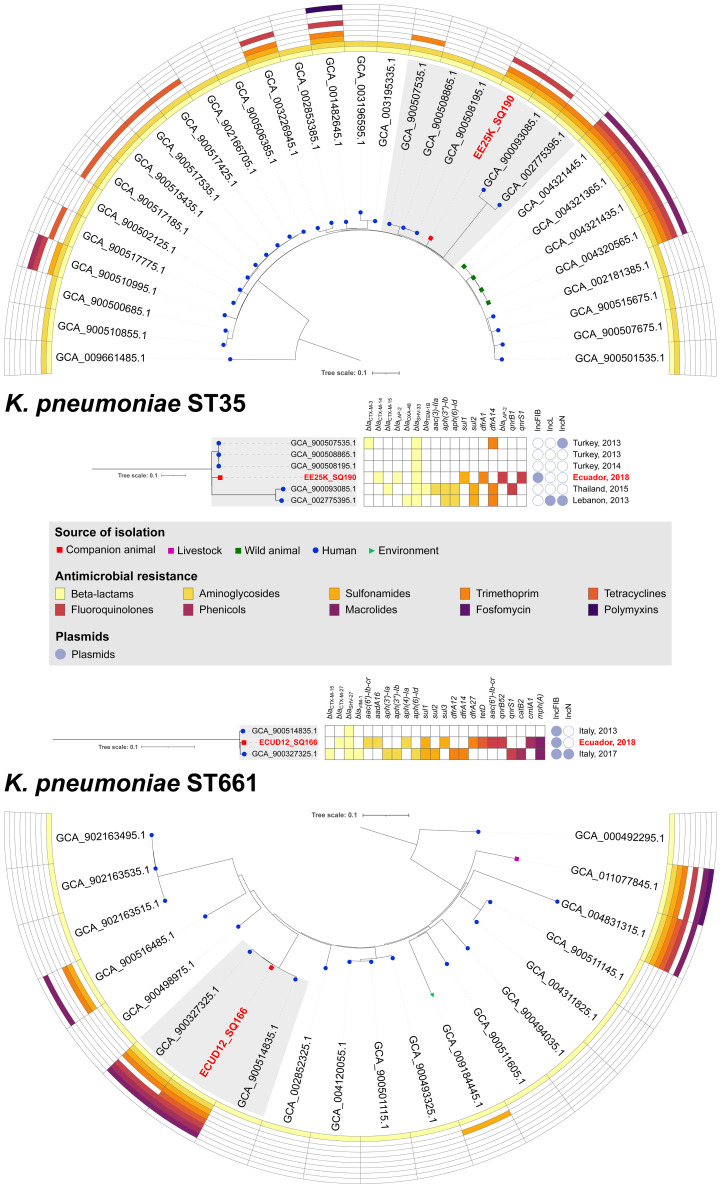
Phylogenetic trees of *K. pneumoniae* strains. SNP-based phylogenetic trees of *K. pneumoniae* ST35 and ST661, and heatmap showing presence/absence of antibiotic resistance genes for 10 antibiotics, and their source of isolation. Details on resistome, plasmidome and origin are showed for clusters formed with CTX-M-14-producing *K. pneumoniae* EE25K_SQ190 and CTX-M-27-producing *K. pneumoniae* ECU12_SQ166 strains, isolated from dogs in Ecuador.

For CTX-M-27-positive *K. pneumoniae* ST661 (ECU12_SQ166), phylogenomic analysis revealed relationship (201-216 SNP differences) with human strains identified in Italy, in 2013 and 2017, respectively (**Figure 2**, **Supplementary Table 3**). Strikingly, all the three isolates within the clade carried an IncFIB-type plasmid. Moreover, ECU12_SQ166 and the human strain isolated in 2017 exhibited an identical MDR profile, sharing *bla*_SHV-27_, *sul1* and *mph(A)* resistance genes. In brief, *K. pneumoniae* ST661 is other global clone identified in Italy, China, England, Brazil, Tunisia, Thailand, Uruguay, Mexico and Taiwan (Yan et al., 2015; Ku et al., 2017; Martin et al., 2017; Patil et al., 2019; Piazza et al., 2019; Sghaier et al., 2019; Furlan et al., 2020; Hassen et al., 2020; Ludden et al., 2020; Leangapichart et al., 2021; Papa-Ezdra et al., 2021; Toledano-Tableros et al., 2021).

In companion animals, ESβL production among *Klebsiella* isolates has been associated with CTX-M-14 and CTX-M-15 variants (Harada et al., 2016). In this study, CTX-M-14-positive *K. pneumoniae* EE25K_SQ190 belonged to ST35. Although this clone has been previously identified in China, Romania, Yemen, Israel, France, Spain and Thailand (Marcade et al., 2013; Cubero et al., 2016; Alsharapy et al., 2020; Frenk et al., 2020; Kong et al., 2020; Shen et al., 2020; Surleac et al., 2020; Zhong et al., 2020; Leangapichart et al., 2021), phylogenomic analysis clustered (353-354 SNP differences) EE25K_SQ190 with a human clone identified in Turkey in 2013 and 2014 (**Figure 2**, **Supplementary Table 3**).

Although, in Ecuador, occurrence of *E. coli* producing ESβL has been reported in pets, chicken, humans, food, vegetables, broiler farms, and river water samples, in Quito (Vinueza-Burgos et al., 2016; Chiluisa-Guacho et al., 2018; Ortega-Paredes et al., 2018; Ortega-Paredes et al., 2019; Vinueza-Burgos et al., 2019; Zurita et al., 2019; Ortega-Paredes et al., 2020a; Ortega-Paredes et al., 2020b; Zurita et al., 2020), and in other cities such as Guayaquil (Soria Segarra et al., 2018), Esmeraldas (Hedman et al., 2019), Loja (Delgado et al., 2016), and Cuenca (Zurita et al., 2013); as well as in the provinces of Tungurahua and Cotopaxi (Sánchez-Salazar et al., 2020), genomic data are scarce. Specifically, while CTX-M-55 and CTX-M-65-producing *E. coli* have been previously reported in dogs in central Ecuador, and in Quito (Ortega-Paredes et al., 2019; Albán et al., 2020; Salinas et al., 2021), CTX-M-producing *K. pneumoniae* have been isolated from human hosts in Cuenca (Nordberg et al., 2013), Quito, Guayaquil, and Azogues (Zurita et al., 2013), so far.

In summary, we report genomic data of global One Health-associated clones of CTX-M-55 and CTX-M-65-producing *E. coli*, and CTX-M-14 and CTX-M-15-producing *K. pneumoniae* in dogs from the province of Imbabura, in Ecuador. The implementation of a national epidemiological surveillance program is necessary to establish future strategies to control the dissemination of antibiotic-resistant priority pathogens using a One Health approach.

## Data availability statement

The datasets presented in this study can be found in online repositories. The names of the repository/repositories and accession number(s) can be found in the article/supplementary material.

## Ethics statement

The animal studies were approved by MSc. Elena Dorothea Balarezo Cisneros President of the Ethics Committee for Research Processes Yachay Tech University. The studies were conducted in accordance with the local legislation and institutional requirements. Written informed consent was obtained from the owners for the participation of their animals in this study.

## Author contributions

FAG-Z: Conceptualization, Formal analysis, Project administration, Supervision, Writing – original draft, Writing – review & editing. JT: Formal analysis, Writing – review & editing. JV-B: Formal analysis, Methodology, Writing – original draft, Writing – review & editing. MV-B: Investigation, Methodology, Writing – review & editing. AC-A: Methodology, Writing – review & editing. FE: Formal analysis, Methodology, Software, Writing – review & editing. QM: Formal analysis, Methodology, Writing – review & editing. BF: Methodology, Writing – review & editing. ES: Formal analysis, Methodology, Software, Writing – review & editing. JGMP: Formal analysis, Methodology, Validation, Writing – review & editing. MJ: Investigation, Writing – review & editing. NL: Conceptualization, resources, Formal analysis, Writing – original draft, Writing – review & editing.
