# Supplementary material for: Pandemic one health clones of Escherichia coli and Klebsiella pneumoniae producing CTX-M-14, CTX-M-27, CTX-M-55 and CTX-M-65 ESβLs among companion animals in northern Ecuador
Source: Front Cell Infect Microbiol. 2025 Jan 7;13:1259764. doi: 10.3389/fcimb.2023.1259764 (PMC11747428; doi:10.3389/fcimb.2023.1259764)
Supplement: Supplementary file 1 [file DataSheet_1.pdf]

Antibiogram ot the forty-tree cephalosporin-resistant Gram-negative bacteria

										amoxicillin-clavulanic acid	ceftazidime	cefotaxime	ceftriaxone	cefepime	cefoxitin	aztreonam	ertapenem	meropenem	imipenem	nalidixic acid	ceftiofur	ciprofloxacin	Trimethoprim/ sulfamethoxazole	Amikacin	Gentamicin	Enrofloxacin	Chloramphenicol
WGS Sequencing	CODE	ID	SPECIE	CRO	POL B	LAC+	LAC-	PURA	CR/A	AMC	CAZ	CTX	CRO	CPM	CFO	ATM	ETP	MER	IMP	NAL	CT	CIP	SXT	AMI	GEN	ENR	CHL
	**Healthy Apr-Jun2018**																										
	ECU	**2**	***K. pneumoniae***	1		1		1		R	R	R	R	R	S	R	R	R	R		R	R	R	R	R		
ECU3_SQ178	ECU	**3**	***E. coli***	1		1		1		S	R	R	R	R	S	R	S	S	S	R		R	S	S	S	R	R
	ECU	**5**	***E. coli***	1		1		1		S	R	R	R	R	S	R	S	S	S	R		R		S	R		
	ECU	**17**	***E. coli***	1		1		1		S	R	R	R	R	S	S	S	S	S	R		R		S	S		
	ECU	**18**	***E. coli***	1		1		1		S	R	R	R	R	S	R	S	S	S		R	R	R	S	S		
	ECU	**19**	***E. coli***	1		1		1		R	R	R	R	R	R	R	S	S	R			S	S	S	S		
	ECU	**20**	***E. coli***	1		1		1		R	R	R	R	R	S	R	S	S	S		R	R	R	R	R		
	ECU	**21-I**	***Acineto/Enterobacter***	1		1		1		S	R	R	R	R	S	R	S	S	R		R	S	S	S	S		
	ECU	**21-II**	***K. pneumoniae***	1		1		1		R	R	R	R	R	S	R	S	S	S		R	R	R	S	R		
	ECU	**22-I**	***E. coli***	1		1		1		S	R	R	R	R	S	R	S	S	S		R	S	S	S	S		
	ECU	**24**	***E. coli***	1		1		1		S	R	R	R	R	S	R	S	S	S		R	R	S	R	S		
	**Sick Apr-Jun2018**																										
	ECU-D	**1**	***K. pneumoniae***	1		1		1		R	R	R	R	R	S	R		R	R		R	R	R	R	R		
	ECU-D	**4**	***E. coli***	1		1		1		R	R	R	R	R	R	R	S	S	S		R	S	R	S	S		
	ECU-D	**12**	***E. coli***	1		1		1		R	R	R	R	R	S	R	S	S	S		R	S	R	S	S		
ECUD12_SQ166	ECU-D	**12**	***K. pneumoniae***	1		1		1		R	R	R	R	R	S	R	S	S	S	R	R	R	R	S	R	R	
	ECU-D	**15**	***E. coli***	1		1		1		R	R	R	R	R	S	R	S	S	S		R	R	R	S	R		
	ECU-D	**18**	***Acineto/Enterobacter***	1		1		1		S	R		R	R	S	R	S	S	S		R	S	R		R		
	ECU-D	**18**	***E. coli***	1		1		1		R	R		R	R	?	R	S	S	S		R	R	R	R	R		
	ECU-D	**19**	***Acineto/Enterobacter***	1		1		1		S	R		R	R	S	R	S	S	S		R	S	S	S	S		
	ECU	**15**	***E. coli***	1		1		1		R	S	R	R	R	R	R	S	S	R		R	R	S	S	S		
	**Healthy Oct-Dec2018**																										
	ES	**5**	***E. coli***	1		1		1		S	S	R	R	R	S	R	S	S	S	S		S	S	S	S		
	ES	**9**	***E. coli***	1		1		1		R	S	R	S	S	R	S	S	S	S	S		S	S	S	S		
	ES	**12**	***E. coli***	1		1		1		S	R	R	R	R	S	R	S	S	S	R		R	S	S	S		
	ES	**17**	***E. coli***	1		1		1		R	R	R	R	R	R	R	R	S	R	S		S	S	S	S		
	ES	**18**	***E. coli***	1		1		1		R	R	R	R	R	R	R	S	S	S	S		S	S	S	S		
	ES	**19**	***E. coli***	1		1		1		S	R	R	R	R	S	R	S	S	S	R		R	R	S	S		
	ES	**25**	***E. coli***	1		1		1		S	R	R	R	R	S	R	S	S	S	R		R	R	S	R		
	ES	**26**	***E. coli***	1		1		1		R	R	S	R	S	R	S	S	S	S	S		S	R	S	S		
	ES	**27**	***E. coli***	1		1		1		R	R	R	R	S	R	R	S	S	S	S		S	R	S	S		
	ES	**30**	***E. coli***	1		1		1		S	R	R	R	R	S	R	S	S	S	S		S	S		R		
	ES	**31**	***E. coli***	1		1		1		S	R	R	R	R	S	R	S	S	S	S		S	S	S	S		
	ES	**34**	***E. coli***	1		1		1		S	R	R	R	R	S	R	S	S	S	S		S	S	S	S		
	**Sick Oct-Dec2018**																										
	EE	**4**	***E. coli***	1		1		1		S	R	R	R	S	S	S	S	S	S	R		R	R	E	R		
	EE	**2**	***E. coli***	1		1		1		S	R	R	R	R	S	R	S	S	S	R		R	R	S	R		
EE12_SQ154	EE	**12**	***E. coli***	1		1		1		S	R	R	R	R	S	R	S	S	S	R		R	R	S	R	R	R
	EE	**13**	***E. coli***	1		1		1		S	R	R	R	R	S	R	S	S	S	S		S	R	S	S		
	EE	**14**	***E. coli***	1		1		1		S	R	R	R	R	R	R	S	S	S	S		S	R	S	S		
	EE	**16**	***E. coli***	1		1		1		R	S	R	R	S	R	R	S	S	R	S		S	R	S	S		
	EE	**21**	***E. coli***	1		1		1		S	R	R	R	R	S	R	S	S	S	R		R	R	S	R		
	EE	**25C**	***E. coli***	1		1		1		R	R	R	R	S	R	R	S	S	R	S		S	S	S	S		
EE25K_SQ190	EE	**25K**	***K. pneumoniae***	1		1		1		S	S	R	R	R	S	S	S	S	S	R		R	R	S	S	R	
	EE	**29**	***E. coli***	1		1		1		S	R	R	R	R	S	R	S	S	S	R		R	R	S	S		
	EE	**33**	***E. coli***	1		1		1		S	R	R	R	R	S	S	S	S	S	R		R	R	S	R		
