# Supplementary figures and images for: Pandemic one health clones of Escherichia coli and Klebsiella pneumoniae producing CTX-M-14, CTX-M-27, CTX-M-55 and CTX-M-65 ESβLs among companion animals in northern Ecuador
Source: Front Cell Infect Microbiol. 2025 Jan 7;13:1259764. doi: 10.3389/fcimb.2023.1259764 (PMC11747428; doi:10.3389/fcimb.2023.1259764)

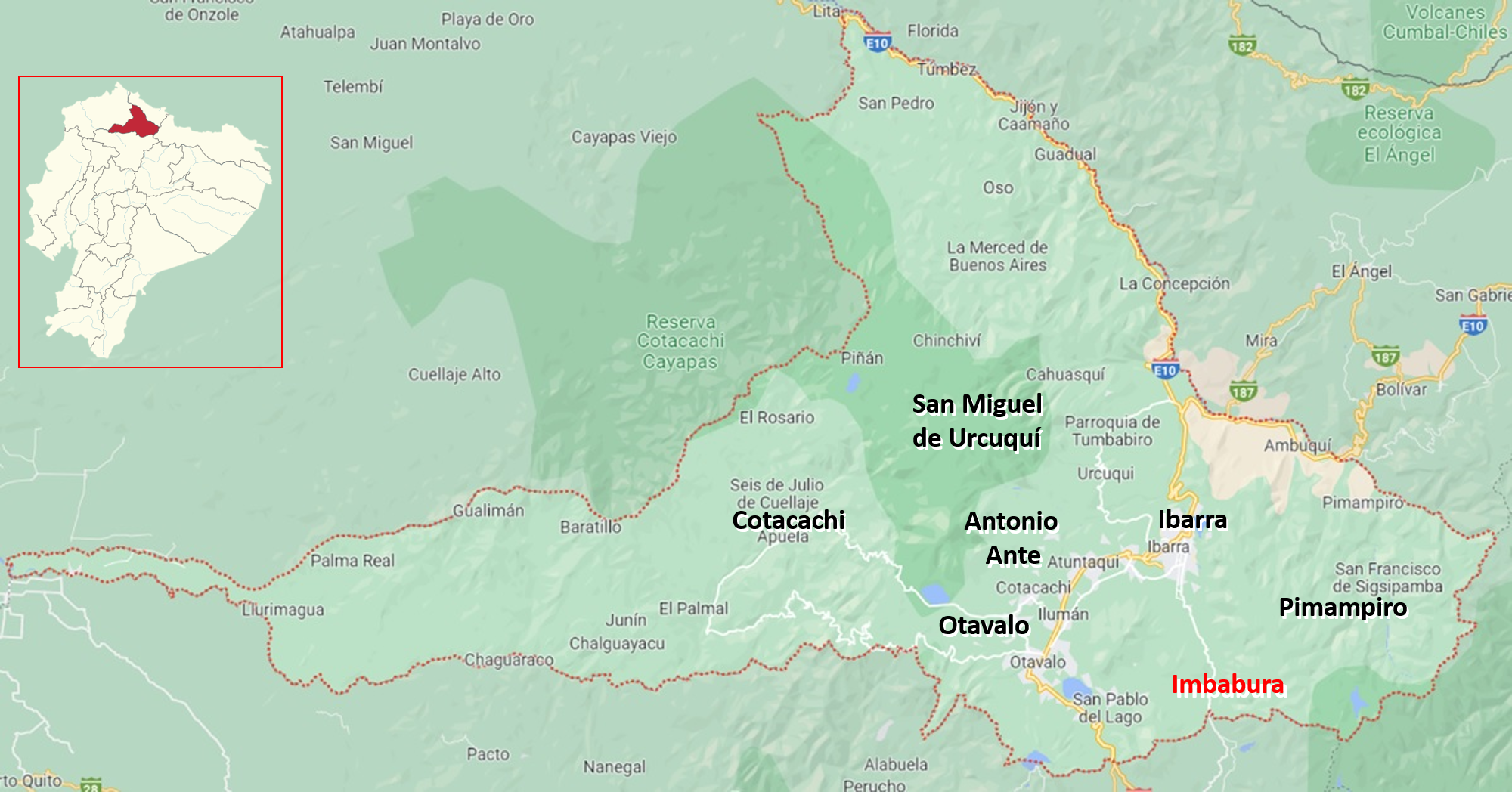

Supplement: Supplementary file 2 [file Image_1.tif]
